# The *Populus* Superoxide Dismutase Gene Family and Its Responses to Drought Stress in Transgenic Poplar Overexpressing a Pine Cytosolic Glutamine Synthetase (GS1a)

**DOI:** 10.1371/journal.pone.0056421

**Published:** 2013-02-22

**Authors:** Juan Jesús Molina-Rueda, Chung Jui Tsai, Edward G. Kirby

**Affiliations:** 1 Department of Biological Sciences, Rutgers University, Newark, New Jersey, United States of America; 2 Warnell School of Forestry and Natural Resources and Department of Genetics, University of Georgia, Athens, Georgia, United States of America; Instituto de Biología Molecular y Celular de Plantas, Spain

## Abstract

**Background:**

Glutamine synthetase (GS) plays a central role in plant nitrogen assimilation, a process intimately linked to soil water availability. We previously showed that hybrid poplar (*Populus tremula X alba*, INRA 717-1B4) expressing ectopically a pine cytosolic glutamine synthetase gene (GS1a) display enhanced tolerance to drought. Preliminary transcriptome profiling revealed that during drought, members of the superoxide dismutase (SOD) family were reciprocally regulated in GS poplar when compared with the wild-type control, in all tissues examined. SOD was the only gene family found to exhibit such patterns.

**Results:**

*In silico* analysis of the *Populus* genome identified 12 *SOD* genes and two genes encoding copper chaperones for SOD (*CCSs*). The poplar SODs form three phylogenetic clusters in accordance with their distinct metal co-factor requirements and gene structure. Nearly all poplar *SODs* and *CCSs* are present in duplicate derived from whole genome duplication, in sharp contrast to their predominantly single-copy *Arabidopsis* orthologs. Drought stress triggered plant-wide down-regulation of the plastidic copper SODs (CSDs), with concomitant up-regulation of plastidic iron SODs (FSDs) in GS poplar relative to the wild type; this was confirmed at the activity level. We also found evidence for coordinated down-regulation of other copper proteins, including plastidic CCSs and polyphenol oxidases, in GS poplar under drought conditions.

**Conclusions:**

Both gene duplication and expression divergence have contributed to the expansion and transcriptional diversity of the *Populus SOD/CCS* families. Coordinated down-regulation of major copper proteins in drought-tolerant GS poplars supports the copper cofactor economy model where copper supply is preferentially allocated for plastocyanins to sustain photosynthesis during drought. Our results also extend previous findings on the compensatory regulation between chloroplastic CSDs and FSDs, and suggest that this copper-mediated mechanism represents a common response to oxidative stress and other genetic manipulations, as in GS poplars, that affect photosynthesis.

## Introduction

Inorganic nitrogen (N) is the most limiting nutrient affecting the growth of forest trees. As N uptake is influenced by soil water availability [1], [2], this problem is exacerbated by increasingly frequent episodes of drought in many regions of the world due to ongoing climate change [3]. In addition to the adverse effects on mineral nutrient uptake, drought causes oxidative stress in plants, including poplar [4], [5]. As such, the drought stress response is tightly coupled with the antioxidant defense system and cellular redox regulation [6].

Glutamine synthetase (GS) plays a central role in assimilation of ammonium into amino acids and other reduced N compounds in plants. Consistent with the central importance of N metabolism in plant growth and development, hybrid poplar (*Populus tremula X alba*, INRA 717-1B4) expressing ectopically the pine glutamine synthetase gene (GS1a) exhibited several pleiotropic phenotypes of agronomic significance. These include increased growth [7], [8], increased nitrogen use efficiency [9], altered wood chemistry [10], and of particular relevance to the present investigation, enhanced tolerance to drought [11].

The superoxide dismutases (SODs) constitute a first line of defense against reactive oxygen species (ROS) [12]. SODs are metalloenzymes that catalyze the dismutation of ion superoxide into oxygen and hydrogen peroxide [13]. The superoxide radical is a ROS whose production increases under abiotic and biotic stresses, including drought [14]. Thus, SODs play a critical role in protecting plant tissues from ROS [12]. SODs are classified according to their metal cofactors and/or subcellular distribution. The predominant forms of SOD in plants are mitochondrial manganese SODs (MnSODs), cytosolic copper/zinc SODs (Cu/ZnSODs), chloroplastic Cu/ZnSODs, and iron SODs (FeSODs) [15]. In addition, plant SODs have been localized in peroxisomes, glyoxysomes [16], vacuoles, the nucleus [17], and the extracellular matrix [18]. Expression of plant SOD genes is regulated by developmental and environmental cues, including hormones [19], [20], high light and UV [15], and drought [21]. Recent work at the molecular level has shown that SOD expression can be modulated by alternative splicing [18], [22] and microRNAs [23], [24]. Transgenic plants that over-express SOD genes display a range of phenotypes depending on the targeted SOD isoform, the level of transgene expression, and subcellular localization. Reported phenotypic effects include enhanced tolerance to oxidative stresses, such as drought and salinity [25]–[27].

Considering the relevant role of the SODs in drought tolerance, we have undertaken *in silico* characterization of the SOD gene family in poplar and assessed transcript levels for the SOD gene family in various tissues of GS transgenic and wild type poplars subjected to drought treatments. Furthermore, we have detected the activities of the major poplar SODs in gel assays. Our results show that drought tolerant GS poplars have altered SOD expression when compared with the wild type under drought conditions. The putative roles of the poplar SOD gene family and the use of specific SODs as marker(s) of drought tolerance are proposed.

## Materials and Methods

### Plant Materials and Stress Treatments

Hybrid poplar (*Populus tremula X P. alba*, INRA 717-1B4) expressing ectopically the pine glutamine synthetase gene (GS1a) were generated and maintained as previously described [7]. Water stress treatments and conditions of recovery from water stress were as described in El-Khatib et al. [11]. Rooted cuttings (9–12 months old) were planted in 6-inch pots containing a peat-based commercial growth medium (Metro-Mix 200, Scotts, Marysville, OH) without supplementary nutrients and raised in a growth chamber supplying a 16 h photoperiod (24–26°C). Soil samples were weighed after drying overnight at 60°C and volumetric soil moisture contents (θ) were calculated. Nonlinear regression (SigmaPlot v4.01, SPSS, Chicago, IL) was used to relate θ to soil water potential (ψ_soil_): ψ_soil_ = 0.9031+1.305 ln(θ–0.1081) (R2 = 0.98; P<0.0001). This allowed conversion of θ, estimated with a time-domain-reflectrometry (TDR) soil moisture meter (Theta Meter, Delta-T Devices, Cambridge, U.K.), to track changes in soil water throughout the experiment. We used soil water potential as a proxy measure of plant water status. Plants were watered every day until θ was between 50 and 55%, equivalent to a soilwater potential of –1 to 0 MPa for well-watered conditions. Drought stress was applied to plants by withholding irrigation for 7 days, by which time θ was between 15 and 20%, equivalent to a soil water potential of –2 to–3 MPa. This level of water stress typically resulted in a decline in leaf stomatal conductance in wild type poplars from 0.138 mol m^−2^s^−1^ (SE 0.025) for well-watered leaves to 0.018 mol m^−2^s^−1^ (SE 0.002) during drought conditions (unpublished data). After the drought treatment, plants were watered every day for 5 days recovering the well-watered conditions in soil. Plants heights ranged from 45 to 55 cm at the collection day.

### Sequence Analysis

Published *Arabidopsis* and *Populus* SODs (NCBI) were used to search the *P. trichocarpa* genome v2.2 (www.phytozome.net) by BLAST [28]. Open reading frames, exon-intron predictions, and 3′-UTRs were manually examined and analyzed against publicly available poplar ESTs. Theoretical molecular weights and isoelectric points for the predicted proteins were calculated using the Expasy server (http://expasy.org/tools/pi_tool.html) [29]. Pairwise sequence similarities were calculated individually using the EBI EMBOSS Pairwise Sequence Alignment server (http://www.ebi.ac.uk/Tools/emboss/align/). The similarity of a group was calculated as the mean of all individual pairwise comparisons within that group. The similarity between groups was calculated as the mean of all between-group pairwise comparisons.

**Figure 1:**
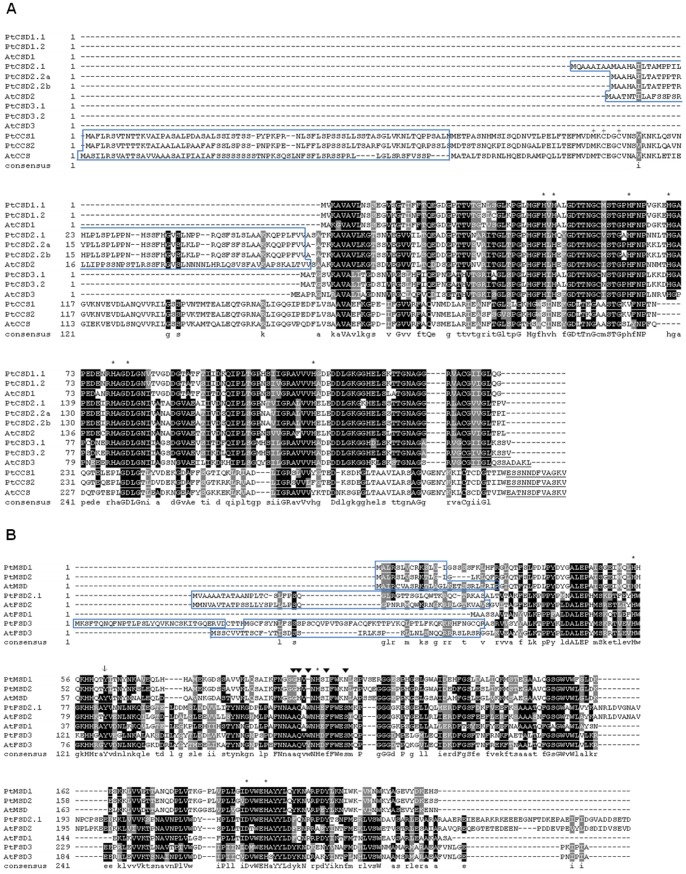
Alignment of predicted SOD and CCS amino acid sequences from *Populus trichocarpa* and *Arabidopsis thaliana*. Blue boxes in the amino termini and underlined sequences in carboxy termini represent predicted transit peptides (see Table 1 for details). Alignments were generated using ClustalX 2.0.12 [30]. Boxes showing identical (black) and similar (grey) amino acids and the consensus sequence were included in the alignment by Boxshade 3.21 (www.ch.embnet.org/software/BOX_form.html). **A.** CSD and CCS alignment. Amino acids involved in copper binding for CCSs in the consensus region MXCXXC [51] are marked with pluses (+) in their amino termini. Amino acids involved in metal binding for CSD group [52] are marked with asterisks (*). **B**. MSD and FSD alignment. Metal ligands [53] and the tyrosine residue essential for catalytic activity [22] are marked with asterisks (*) and tail arrows (**↓**), respectively. The primary candidates for distinguishing MSD from FSD [72] are indicated with solid arrowheads. Tryptophan residues within this region may confer H_2_O_2_ sensitivity in FSDs [73].

The alignments in Figure 1 were prepared using ClustalX 2.0.12 [30]. Boxshade 3.21 (www.ch.embnet.org/software/BOX_form.html) was used to mark identity and similarity boxes and consensus lines in amino acid alignments. The Neighbor-joining tree was constructed using the Muscle alignment program implemented in MEGA version 5 [31], with partial deletion to handle alignment gaps, and 1000 bootstrap iterations. Poplar SOD gene nomenclature in this paper was assigned considering its phylogenetic relationship with the published nomenclature for the *Arabidopsis* SOD gene family [15].

TargetP 1.1 [32] (http://www.cbs.dtu.dk/services/TargetP/) was used for general subcellular localization prediction of poplar SODs and CCSs. Following the recommendation of Emanuelsson et al. [32], proteins predicted as “other” (other than chloroplast, mitochondria or secreted) by the TargetP 1.1 were further analyzed by TMHMM 2.0 (http://www.cbs.dtu.dk/services/TMHMM/) to assess transmembrane helices. Sequences predicted as “secretory” or had low reliability (RC≥4) were further analyzed using SignalP 4.0 [33] (http://www.cbs.dtu.dk/services/SignalP/). ChloroP 1.1 [34](http://www.cbs.dtu.dk/services/ChloroP/) and MITOPROT [35] were used to produce a detailed report for chloroplast- and mitochondria-targeted proteins, respectively. PTS1 [36] (http://www.mendel.imp.ac.at/mendeljsp/sat/pts1/PTS1predictor.jsp) was used for peroxisomal protein predictions.

### qPCR

RNA extraction was carried out as described in Liao et al. [37]. RNA was extracted from two biological replicates consisting of pooled samples from 5 individual plants from 2 replicate experiments. Each experiment assessed the GS transgenic line (line 4–29) and the wild type control. Quality of the RNA was assessed both on agarose gels and spectrophotometrically. Although no contamination by genomic DNA was detected on gels, all RNA samples were treated with DNases (Turbo DNA Free kit of Applied Biosystems/Ambion, Austin TX), following the manufacturer’s protocol, and stored at −80°C for up to three months. For cDNA synthesis, the iScript Select cDNA Synthesis kit (Bio-Rad, Hercules, CA) was used with both random and oligo dT primers using 3 µg of total RNA per reaction (80 µL), according to the manufacturer’s instructions. cDNAs were stored at −20°C for up to six months.

Quantitative PCR was performed using a LightCycler 480 (Roche Applied Science, Indianapolis IN) using Roche SYBR Green I Master mix prepared according to the manufacturer’s specifications. qPCR reactions were carried out in 20 µL volumes containing 10 ng cDNA and 0.5 µM primers. A total of 45 cycles were run per program: denaturing was at 95°C for 10 sec, annealing at 58°C for 15 sec, and extension was at 72°C for 12 seconds in each cycle.

*P. trichocarpa* genome sequences and *Populus* EST sequences (*P. tremula* and *P. alba*) were used in the design of the primers for qPCR (Table S1). The forward primers were designed within the coding regions and the reverse primers were designed in 3′ UTRs. Primer quality was evaluated using Prime3Plus (www.bioinformatics.nl/cgi-bin/primer3plus/) [38]. All amplicons were between 155 and 305 bp. Sequences of the resulting amplicons were validated by sequencing the RT-qPCR product. Relative transcript levels were determined against three validated reference genes: actin, elongation factor 1β and ubiquitin [39], using GeNorm [40] (Figure S1). Quantitative cycles were estimated using LinRegPCR (v 11.1) [41]. In all cases, two biological replicates were used, each with three technical replicates.

Cluster 3.0 [42] and Java TreeView [43] programs were used as the computational and graphical environment for analyzing correlations from RT-qPCR expression data. The heat map was generated using Heat Mapper Plus (Bio-Array Resource for Plant Biology; http://bar.utoronto.ca/welcome.htm).

### Determination of SOD Activities

In order to provide assessment of qualitative differences in activities of the various SODs in GS transgenic and control leaves, proteins were extracted from three biological replicates (individual plants) in two replicate experiments and on native protein gels. Proteins were extracted by mixing one part of liquid nitrogen-ground tissue with two parts of extraction buffer [50 mM KH_2_PO_4_ pH 7.8, 1 mM EDTA, 0.1% (w/v) Triton X-100, and 0.05% (v/v) β-mercaptoethanol] and incubated on ice for 10 min. Samples were centrifuged at 13,000 g for 12 min at 4°C and protein concentrations were determined spectrophotometrically [44] using BSA as a standard. The protocol of Weydert and Cullen [45] was followed to assess SOD activities using native gels (acrylamide and bis-acrylamide solution (29∶1) 12%, w/v; 1.5 mm thickness) with slight modifications. Gels were first run at 20 mA for one hour, followed by 30 mA for two hours, after which the electrophoresis buffer was replaced. The gels were then run at 40 mA for 20 min after run-off of the dye front. Seventy-five micrograms total protein was found optimal for protein separation. Assays of the three SOD activities (Cu/ZnSODs, MnSODs, and FeSODs) were performed using specific inhibitors (KCN and H_2_O_2_), as previously described [46]. Gels were scanned, negative images were obtained, and intensities of bands were measured using Image J 1.43 [47].

## Results

### *In silico* Characterization of the SOD Gene Family in *Populus*

**Figure 2:**
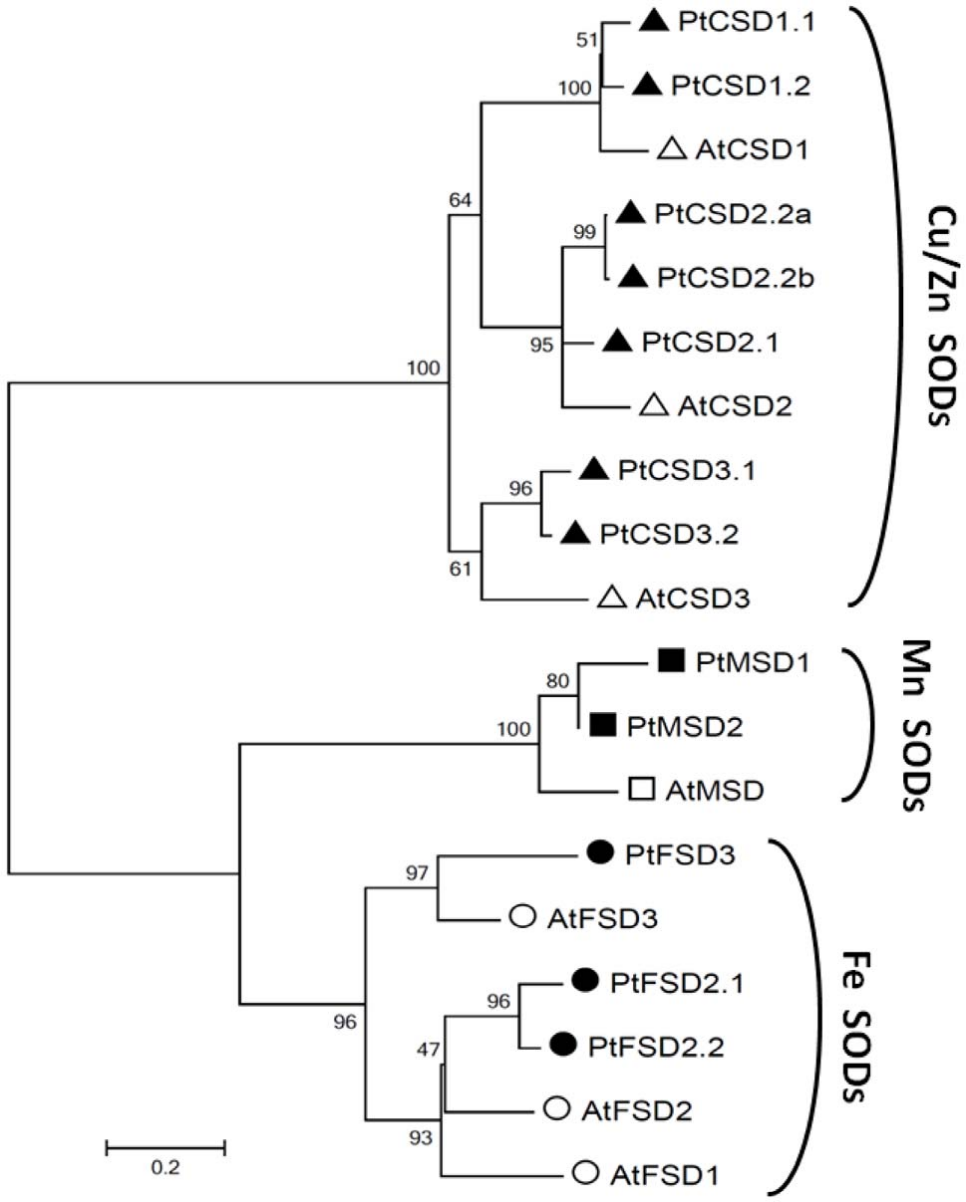
Phylogenetic analysis of *Populus trichocarpa* (Pt) and *Arabidopsis thaliana* (At) SODs based on predicted amino acid sequences. The neighbor-joining tree was generated using Mega 5.05 [31]. The bootstrap method with 1000 replicates was used as a test of the phylogeny. The three groups identified include the copper/zinc SODs (triangles), manganese SODs (squares), and iron SODs (circles). Poplar and *Arabidopsis* sequences are marked with solid and empty symbols, respectively.

Twelve putative SODs were identified in the *P. trichocarpa* genome (Phytozome) by BLAST using *Arabidopsis* and poplar sequences functionally annotated as SODs in the NCBI database as queries. To propose a nomenclature for the poplar SOD gene family, a phylogenetic tree was constructed using predicted amino acid sequences from *Populus* and *Arabidopsis* (Figure 2). *Arabidopsis* is the only plant for which the SOD gene family has been fully characterized [15]. In *Arabidopsis* the SOD family consists of seven members: three Cu/ZnSODs (*AtCSD*s), one MnSOD (*AtMSD*), and three FeSODs (*AtFSD*s). The three groups formed separate clusters in the phylogenetic tree with strong bootstrap support, in accordance with their distinct metal cofactor requirements (Figure 2). Seven poplar SODs were classified as Cu/ZnSODs in three strongly supported sub-groups (PtCSD1, PtCSD2, and PtCSD3) corresponding to their putative *Arabidopsis* orthologs. The PtCSD1 sub-group contains two highly similar isoforms, PtCSD1.1 and PtCSD1.2 (96.1% amino acid sequence similarity, Figure S2), derived from the recent (Salicoid) whole-genome duplication [48] (Plant Genome Duplication Database [http://chibba.agtec.uga.edu/duplication/]). They share high similarity (91–92%) to the putative ortholog, AtCSD1 (Figure S2). The PtCSD2 sub-group contains three SODs, PtCSD2.1, PtCSD2.2a and PtCSD2.2b, two of which are nearly identical (PtCSD2.2a and PtCSD2.2b; 99.5% similarity). PtCSD2.1 and PtCSD2.2b (87.2% similarity) were derived from the Salicoid whole-genome duplication (Plant Genome Duplication Database), whereas PtCSD2.2a likely originated from PtCSD2.2b via an independent duplication event. The PtCSD2s share 75–80% amino acid sequence similarity with the *Arabidopsis* ortholog, AtCSD2. The third sub-group also contains a genome duplicate, PtCSD3.1 and PtCSD3.2, with high similarity with one another (96.2%) and with the *Arabidopsis* AtCSD3 (82–84%).

The MnSOD group is the smallest of the three, with two poplar members, PtMSD1 and PtMSD2 (93.0% similarity), derived from genome-wide duplication. They share 86–87% similarity with their *Arabidopsis* ortholog AtMSD. The FeSOD group contains equal numbers of *Populus* and *Arabidopsis* SODs in two sub-clusters. One poplar isoform grouped with AtFSD3 (66.9% similarity) with very strong bootstrap support, and was designated PtFSD3. The other two were derived from genome-wide duplication; one appeared to be a partial sequence. The full-length isoform (POPTR_0015s12190) was most similar to AtFSD2 (77.5%: Figure S2), thus designated PtFSD2.1, whereas the truncated gene model (POPTR_0012s11400) was named PtFSD2.2. Manual inspection identified five miss-annotated introns and five exons (Figure S3). The curated gene model contained nine exons (versus four in the Phytozome-predicted model), similar to *PtFSD2.1*. However, one of the exons in *PtFSD2.2* harbored two single-nucleotide insertions relative to *PtFSD2.1* (shaded residues in Figure S3), the first of which led to a premature stop codon. This suggests that PtFSD2.2 may represent a pseudogene. The lone member AtFSD1 shares 57% amino acid sequence similarity with AtFSD2, and they were derived from an older, Brassicaceae-specific (β) duplication event (Plant Genome Duplication Database). Consistent with this, no apparent *Populus* ortholog of AtFSD1 was identified.

Copper chaperones for Cu/ZnSODs (CCS) were included in this work, since CCS are required for Cu/ZnSOD activity in *Arabidopsis* [49]. Two putative CCSs homologous to the *Arabidopsis* AtCCS were identified in the *Populus* genome, and were designated PtCCS1 and PtCCS2. They appear derived from whole-genome duplication, and shared 90.7% similarity with each other, and 77–79% with AtCCS (Figure S2). Like several of the SODs, transcript levels of both CCS genes were significantly altered in the GS poplar relative to the wild type under drought, based on our microarray studies (data not shown).

Taken together, our analysis showed that multiple gene duplication events contributed to the expansion of the *Populus* SOD and CCS families. This resulted in the overall greater numbers of poplar genes in each SOD/CCS group than the number of orthologs found in *Arabidopsis*, except for the iron SOD group.

### Gene Structure of *Populus* and *Arabidopsis* SODs and CCSs

**Figure 3:**
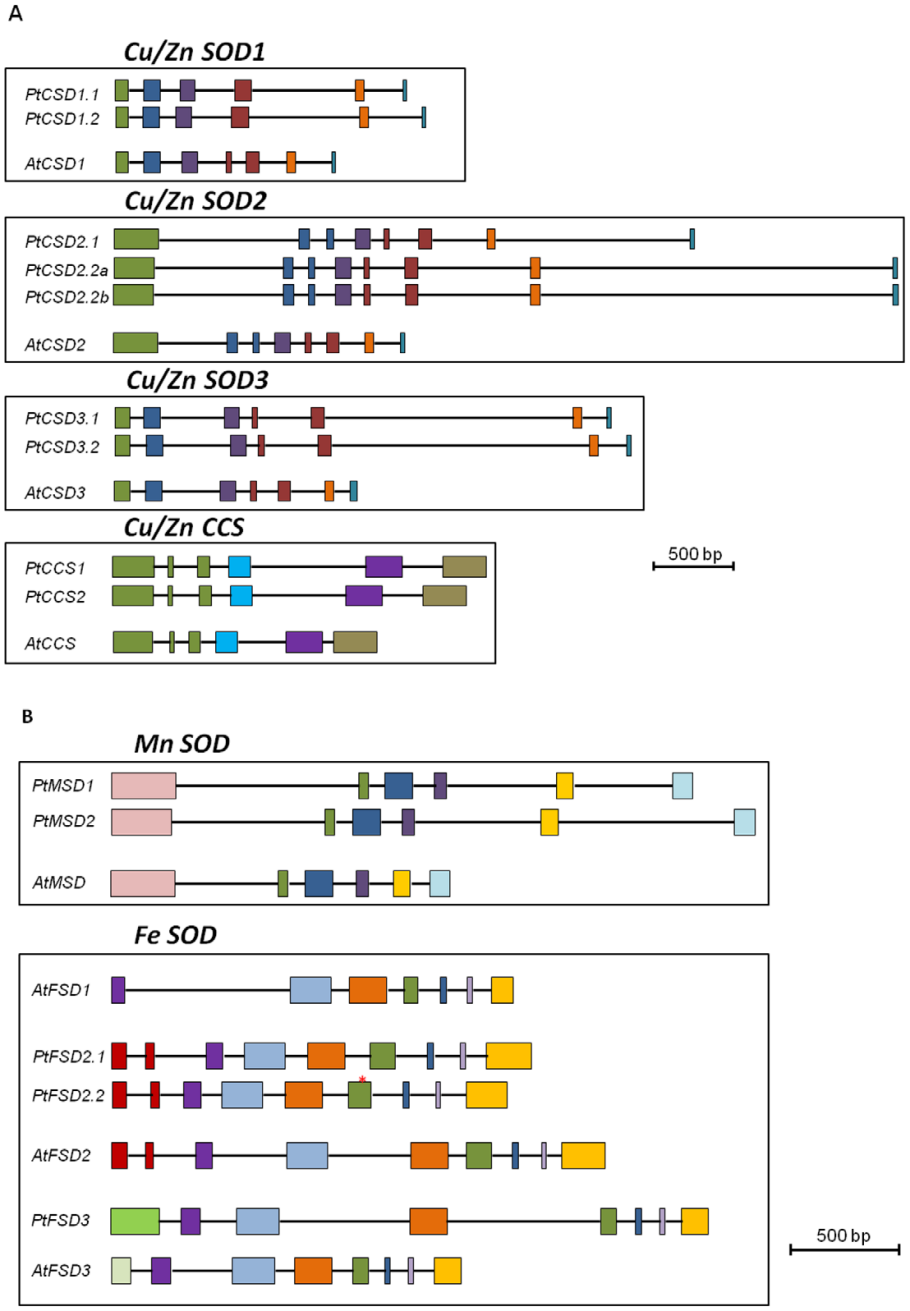
Gene structure (exons and introns) of *Populus trichocarpa* and *Arabidopsis thaliana* SODs and CCSs. **A.** Gene structure for *CSDs* and *CCSs*. **B.** Gene structure for *MSDs* and *FSDs*. Exons, shown with squares, and introns shown as lines, are drawn to scale. Similar or equivalent exons based on similarities in their encoding amino acid sequences have the same color within each group (*CCSs*, *CSDs*, *MSDs* and *FSDs*).

The exon-intron structure was largely conserved among *Populus* and *Arabidopsis Cu/ZnSOD* genes, with two exceptions. The exons 4 and 5 were fused in *PtCSD1.1* and *PtCSD1.2*, whereas the second exon was split into two in the *CSD2* group (Figure 3A). The length of exon 1 in the *CSD2* group is more than twice as long as exon 1 in the other Cu/ZnSOD groups, due to the presence of putative chloroplast targeting sequence (see below). The gene structure of *CCSs* is distinct from that of the *Cu/ZnSODs*, but is conserved between *Populus* and *Arabidopsis* (Figure 3A). The poplar and *Arabidopsis MnSOD* genes have similar structures (Figure 3B). Gene structure conservation between *Populus* and *Arabidopsis* was also observed for the *FeSOD* genes, except for the 5′ region that differed among the subgroups (Figure 3B). The lone *AtFSD1* is the shortest, lacking any putative subcellular targeting sequence (see below), perhaps consistent with its origin from a lineage-specific duplication event. Relative to *FSD1*, *FSD2* genes contain two additional exons, and *FSD3* genes, one, at the 5′-end. Across all SOD/CCS groups, many of the introns were longer in the *Populus* genes than in the *Arabidopsis* homologs, consistent with the genome-wide trend reported earlier [50].

### Conserved Sequence Motifs and Subcellular Localization Prediction

In order to assess conservation of key amino acids for active sites and metal binding domains in the poplar SODs and CCSs, the sequences were divided into two groups for alignment: the Cu/Zn binding group including Cu/ZnSODs and CCSs (Figure 1A), and the manganese and iron binding group (Figure 1B). In both groups, all residues previously shown to be involved in metal cofactor binding [51]–[53] are conserved in the poplar proteins (the truncated PtFSD2.2 was excluded from this analysis).

The N-terminal regions were less conserved in both groups, harboring putative transit peptides for subcellular targeting. Several programs, including TargetP 1.1 (for multi-compartments prediction [32]), ChloroP1.1 (for chloroplastic targeting, [54]), MITOPROT (for mitochondrial prediction [55]), and the PTS1 predictor (for peroxisomal targeting signal prediction [46]), were used to predict subcellular localization (Table 1). Within the Cu/ZnSODs, the CSD2 group with extended N-termini (Figure 1A and Figure 3A) was predicted to be chloroplast-localized (Table 1). Neither the CSD1 nor CDS3 groups possess recognizable transient peptides for chloroplastic or mitochondrial targeting or secretory proteins. The PTS1 predictor indicated a possible peroxisomal localization for PtCSD3.2 and AtCSD3, with some level of uncertainty (termed “twilight zone”, see [36]). Thus, PtCSD3.2 and AtCSD3 were predicted to be cytosolic or have predicted peroxisomal targeting, while PtCSD3.1 and the CSD1 group were predicted to be cytosolic (Table 1). Our predictions for the *Arabidopsis* CSDs are consistent with those reported earlier [15]. The most consistent subcellular prediction for the CCSs was chloroplast, as reported for AtCCS [54]. In addition, the PTS1 predictor classified PtCCS2 and AtCCS as targeted to the peroxisomes, with PtCCS1 receiving a similar prediction in the “twilight zone”. Moreover, the second methionine in the two poplar and the *Arabidopsis* CCSs is conserved, and it has been suggested as a second translational start site from which a cytosolic isoform can be produced [55]. Thus, the CCS proteins were predicted to be either cytosolic, chloroplastic, or peroxisomal (Table 1).

**Table 1:** Predicted characteristics of SOD and CCS amino acid sequences from *Populus trichocarpa* and *Arabidopsis thaliana*.

Populus	Length (a.a.) precursor/mature protein	pI precursor/mature protein	Subcellular prediction	*Arabidopsis*	Length (a.a.) precursor/mature protein	pI precursor/matureprotein	Subcellular prediction
**PtCSD1.1**	152	5.6	Cytosolic	**AtCSD1**	152	5.24	Cytosolic
**PtCSD1.2**	152	5.47	Cytosolic	**AtCSD2**	216/155	6.49/5.30	Chlorop.
**PtCSD2.1**	219/156	6.28/5.49	Chlorop.	**AtCSD3**	164	7.16	Cytosolic, Perox*
**PtCSD2.2a**	210/155	6.39/5.34	Chlorop.	**AtCCS**	320/254	5.60/4.94	Chlorop., Perox. and Cytosolic
**PtCSD2.2b**	210/155	6.44/5.34	Chlorop.	**AtMSD**	231/205	8.47/6.06	Mitoch.
**PtCSD3.1**	158	6.38	Cytosolic	**AtFSD1**	212	6.06	Cytosolic
**PtCSD3.2**	158	6.82	Cytosolic, Perox.*	**AtFSD2**	305/259	4.89/4.52	Chlorop.
**PtCCS1**	323/253	5.04/4.71	Chlorop., Perox.* and Cytosolic	**AtFSD3**	263/222	8.62/5.89	Chlorop.
**PtCCS2**	323/253	5.47/4.87	Chlorop., Perox. and Cytosolic				
**PtMSD1**	229/215	7.24/6.51	Mitoch.				
**PtMSD2**	225/211	6.80/6.21	Mitoch.				
**PtFSD2.1**	307/264	5.10/4.80	Chlorop.				
**PtFSD3**	308/221	8.09/5.25	Chlorop.				

Included are predicted length in amino acids, predicted isoelectric points (pI), and predicted subcellular localizations. Asterisks denote marginal confidence on the peroxisomal prediction.

All members of the MnSOD group were predicted to be localized in the mitochondria (Table 1). The consensus target prediction for the FeSOD2s and FeSOD3s was chloroplast-targeting (Table 1). The lone AtFSD1 member did not show any transient peptide signal, and was therefore predicted to be cytosolic. Similar predictions for the AtFSDs have been reported [15]. In general, the predicted subcellular localizations, pI values, and amino acid sequence lengths for poplar and *Arabidopsis* SOD proteins are similar (Table 1).

### Transcript Levels of *SOD* and *CCS* Genes in Wild Type and GS Transgenic Poplars

**Figure 4:**
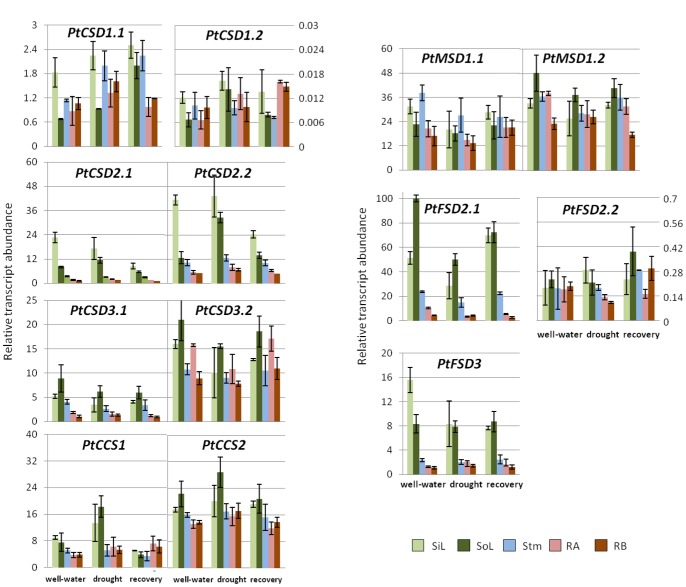
Relative transcript levels of poplar *SODs* and *CCSs* in various tissues under well-watered, drought, and recovery conditions. Transcript levels were measured by RT-qPCR and normalized against three reference genes (see Methods; Figure S1). Sink leaves (**SiL**), source leaves (**SoL**), stems (**Stm**), main roots (**RA**) and fine roots (**RB**) were analyzed. Values represent means of two biological replicates with standard deviations. A two-way ANOVA of observed transcript levels of SOD genes (all tissues vs. water availability) is provided in Table S2.

Transcript levels of the poplar *SOD* and *CCS* genes were investigated using RT-qPCR. Sink leaves, source leaves, young stem, main roots and fine roots from plants subjected to well-watered, drought and drought recovery conditions were analyzed. Transcripts for all genes were detected in all tissues examined, as shown for the wild type in Figure 4, although levels of *PtFSD2.2* and *PtCSD1.2* transcripts were barely detectable (quantification cycles of 30 and 34 in RT-qPCR, respectively), hence they were removed from further analysis. The *PtCSD2s*, *PtFSD2.1* and *PtFSD3* exhibited leaf-biased expression across treatments. *PtCSD2.2* and *PtFSD2.1* were two of the most abundant *SOD* transcripts in our analysis. *PtCSD1.1*, *PtCCSs* and *PtMSDs* showed no clear tissue specificity. The *PtCSD3* pair differed in their tissue distribution patterns, with *PtCSD3.1* transcript levels being higher in green tissues than in roots, and *PtCDS3.2* showing more uniform transcript levels across all tissues (Figure 4).

**Figure 5:**
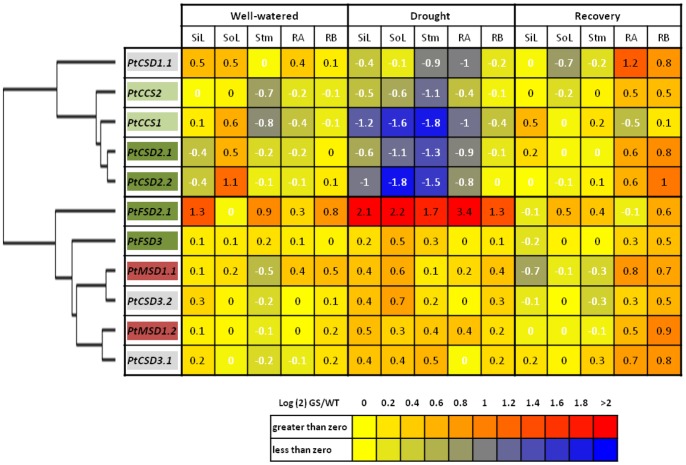
Relative transcript abundance of poplar *SODs* and *CCSs* comparing transgenic GS and wild-type poplars under well-watered, drought or recovery conditions. Values represent the log ratio of transcript levels (transgenics/wild type) (RT-qPCR data; as for Figure 4) for visualization by the Heat Mapper Plus tool (http://bar.utoronto.ca/welcome.htm). Samples are sorted by conditions (well-watered, drought, and recovery) and by tissue [sink leaves (**SiL**), source leaves (**SoL**), stems (**Stm**), main roots (**RA**) and fine roots (**RB**)]. Gene descriptors are colored according to the predicted subcellular localizations (see Table 1) and arranged according to the clustering pattern obtained using the Cluster 3 and Java TreeView programs (see Methods). Genes with significant differences between WT and GS transgenic across tissues under drought stress condition (Table S3) are underlined.

In comparing transcriptional responses to well-watered, drought, and recovery conditions, most *SOD/CCS* genes showed transcriptional responses to drought compared to the well-watered condition (Figure 4 and Table S2). Fewer genes showed significant changes in transcript profiles during recovery when compared with the well-watered condition (Figure 4 and Table S2). In general, greater transcriptional responses were observed in leaves, when compared to other tissues investigated (Figure 4). Likewise, the response due to GS-overexpression was weak when compared with the wild type under well-watered or recovery conditions (Figure 5 and Table S3). However, drought stress triggered considerable differences in transcript levels of *SOD/CCS* genes between wild type and GS poplars (Figure 5 and Table S3). Cluster analysis revealed two distinct expression patterns (Figure 5). One group, consisting of *PtCSD1.1, PtCSD2s* and *PtCCSs*, showed a clear trend of lower transcript abundance in GS transgenics than in the wild type during drought. The second group consisting of *PtCSD3s*, *PtMSDs* and *PtFSDs*, showed the opposite trend: increased expression in GS transgenics. Consistent with the microarray findings (Figure S4), the response of *PtFSD2.1* (up-regulation in GS poplar) and *PtCSD2s* (down-regulation in GS poplar) was particularly notable and wide-spread among tissues.

### Altered SOD Activities in Drought-stressed GS Poplar

**Figure 6:**
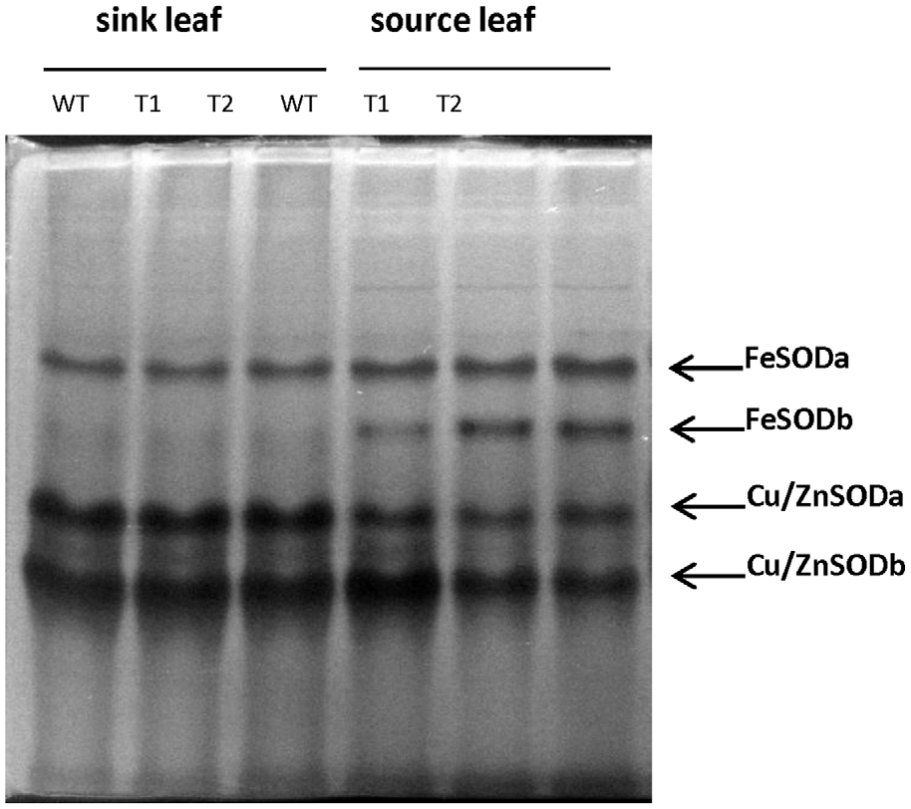
SOD activities as detected by in-gel assays. Total proteins were extracted from source and sink leaves of two transgenic lines (T1 and T2) and wild type control plants (WT) grown under drought conditions. A total of 75 µg protein was loaded per well. Iron and Cu/Zn SOD proteins were identified using specific inhibitors as described by Fridovich [46].

SOD activities were determined by in-gel assays using proteins isolated from leaves of wild type and two GS transgenic lines (Figure 6). Four main bands showing SOD activity were detected. By using specific inhibitors [46], two bands were confirmed as showing FeSOD activity (FeSODa and FeSODb) and two bands showed Cu/ZnSOD activity (Cu/ZnSODa and Cu/ZnSODb). No consistent differences were observed in SOD activity between transgenic and wild type plants under well-watered conditions (data not shown), but significant differences in SOD activities were detected in drought-stressed source leaves of GS transgenic vs. wild type (Figure 6). FeSODb, Cu/ZnSODa and Cu/ZnSODb activities in source leaves were significantly different between transgenic and the wild type control (P<0.05; two-way ANOVA), with activity of the iron SOD higher in GS transgenic leaves than in the wild type (43% increase) while the Cu/Zn SOD a and b activities decreased (38% and 46% decrease respectively). These results are in line with the transcript-level response. Taken together, SOD transcript and protein activity assays support the initial microarray observation that some Cu/ZnSOD and FeSOD members exhibited differential expression responses to GS transgenic manipulation under drought conditions.

## Discussion

The *Populus* genome contains two *CCS* and 12 *SOD* genes, including all major groups of SODs (Cu/ZnSOD, MnSOD and FeSOD) conserved in plants [15]. Relative to *Arabidopsis*, the *Populus CCS/SOD* families are about twice as large, due to duplication in all but one gene (*FSD3*). This is in sharp contrast to the predominantly single-copy nature of the *Arabidopsis CCS/SOD* orthologs (except *AtFSD1*), even though *Arabidopsis* has experienced two rounds of recent (α and β) whole-genome duplication versus one (Salicoid duplication) in *Populus* [56]. The preferential duplicate retention of essentially the entire complement of *SODs* and *CCSs* in *Populus* may hint at their importance in the response of woody perennials to oxidative stress. While expression of some duplicates, e.g., *PtCSD2s* and *PtMSDs*, remained similar in the tissues examined, patterns of transcript distribution of the other SOD pairs appeared to have diverged. For example, transcript levels of *PtCSD3.2* were more evenly distributed across tissues, whereas *PtCSD3.1* exhibited a biased expression in green tissues. In many cases, transcript levels, rather than tissue distribution patterns *per se*, have diverged between duplicate genes, with one copy showing higher expression than the other. The most notable examples are *PtCSD1s*, *PtCSD3s*, *PtCCSs*, and *PtFSD2s.* In the case of the *PtFSD2* pair, the poorly expressed copy (*PtFSD2.2*) is predicted to encode a truncated protein. This suggests that *PtFSD2.2* might have undergone pseudogenization following duplication, and may no longer be functional. Together, our data provide evidence that gene duplication/retention and, in some cases, differential regulation of duplicates have both contributed to the expansion and transcriptional diversity of the *Populus SOD/CCS* families, especially under stress conditions.

Transcript levels were highest for the chloroplast-localized SOD isoforms, e.g., *PtCSD2s*, *PtCCSs*, and *PtFSD2.1*, and these isoforms were also the ones that differed the most between GS poplar and the wild type under drought (Figures 4 and 5). Interestingly, the *PtCSD2/PtCCS* and *PtFSD2.1* genes showed opposite patterns in response to drought, with the *PtCSD2/PtCCS* groups strongly down-regulated, and *PtFSD2.1* up-regulated in GS poplar relative to the wild type. Down-regulation of plastidic *CSDs* with concomitant up-regulation of plastidic *FSDs* has also been reported in a number of species grown under Cu-limiting conditions [57]–[59]. It was suggested that suppression of Cu/ZnSOD during Cu-deficiency allows allocation of the Cu cofactor to plastocyanin, a major Cu-containing protein in the stroma, in order to sustain photosynthesis [54]. In *Arabidopsis*, this model was further supported by coordinated down-regulation of *AtCCS* in response to Cu-limitation [54]. Simultaneous induction of plastidic *FeSOD* is thought to protect chloroplasts against oxidative damage [57], as has been frequently reported in plants [60], [61]. In the case of GS poplars, net photosynthetic rates and chlorophyll contents were higher relative to the wild type, both before and during drought [7], [11]. This is consistent with an increased demand of Cu cofactor for photosynthetic electron transfer, and may occur at the expense of Cu/ZnSOD expression and protein accumulation, as observed in GS poplars. Thus, our results suggest that the Cu-modulated compensatory regulation between chloroplastic Cu/ZnSOD and FeSOD may be a common response to oxidative stress or transgenic manipulations that affect the photosynthesis.

The cytosolic *CSD1* and plastidic *CSD2* and *CCS* are known to be regulated by microRNA 398 (*miR398*) [23], [49]. Although miRNAs were not investigated in the present study, stimulation of poplar *miR398s* by drought may be expected based on the strong down-regulation of their predicted targets, *PtCSD1s*, *PtCSD2s* and *PtCCSs* [62], [63], as has been reported for *Medicago* [64]. Another important yet relatively less emphasized role of *miR398* is its involvement in the regulation of Cu homeostasis [65]. *miRNA398* itself is negatively regulated by Cu, and its predicted targets, *CDS1*, *CDS2*, *CCS* and *COX5b* (mitochondrial cytochrome c oxidase subunit 5b) are Cu-containing proteins [49], [65]. Because metal homeostasis is closely coupled to cellular redox status and antioxidant defense, Yamasaki et al. [65] proposed that *miR398* may be involved in the regulation of copper homeostasis.

The above analysis suggests that enhanced drought resistance of the GS poplars may involve altered Cu homeostasis and miRNA regulation. In addition to the *miR398* targets (*PtCSD1s*, *PtCSD2s* and *PtCCSs*), several chloroplast-localized polyphenol oxidases (PPOs), another major Cu protein family in poplar [66], were down-regulated in GS poplars (Figure S4). *Populus PPOs* were recently shown to be Cu-regulated by a new Cu-responsive miRNA, *miR1444* [66]. The concept of coordinated down-regulation of major Cu proteins (CSD1, CSD2, CCS and PPO) by Cu-responsive *miR398* and *miR1444* is consistent with the Cu cofactor economy model in which Cu is diverted to plastocyanins, thus sustaining the increased photosynthetic rates observed in GS poplars [11]. Interestingly, *miR398* was also found to be regulated by nutrient deficiencies, including N [67]. Taken together, our results suggest that, as a result of altered N metabolism and enhanced photosynthesis, drought tolerance in the GS poplars involves Cu- and miRNA-mediated antioxidant regulation.

*SOD* expression has also been reported to be regulated by ethylene. Kurepa et al. showed that ACC treatment of tobacco leaves increased transcript levels of an iron SOD and decreased transcript levels of a copper SOD [19]. GS poplars show higher levels of glutamine and glutamate, as well as γ-amino butyric acid (GABA) ([9] and data not shown). GABA is a non-proteinogenic amino acid often induced under biotic and abiotic stress conditions [68]. Kathiresan et al. reported that GABA stimulates ethylene biosynthesis in sunflower leaves [69]. Furthermore, glutamate decarboxylase, the principle enzyme in GABA biosynthesis, and ACC synthase and ACC oxidase show highly correlated expression patterns in pine [70]. Transcription of jasmonate-related genes is also affected by ectopic expression of GS in poplar tissues (manuscript in preparation). Thus, the present study shows that enhanced drought tolerance observed in GS poplars is accompanied by differential *SOD* gene expression patterns (i.e. higher iron SOD and lower Cu/Zn SOD expression) and suggests a relationship between GS expression and altered hormone homeostasis and GABA metabolism.

### Conclusions

The *SOD/CCS* families are significantly expanded in *Populus* relative to *Arabidopsis*, although both species have experienced independent rounds of whole genome duplication since they last shared a common ancestor. All but one of the *SOD/CCS* genes retained duplicated copies following whole genome duplication in *Populus*, while only one such pair was retained in *Arabidopsis*. Expression analysis revealed that some of the *Populus* paralogs have already diverged in their transcript abundance, tissue distribution patterns and/or stress response. We observed a coordinated down-regulation of the plastidic PtCDS2s and up-regulation of the plastidic PtFSDs, at the mRNA as well as activity levels, in drought-stressed GS transgenics. This is consistent with preferential allocation of Cu cofactor to plastocyanin to sustain high rates of photosynthesis in the GS transgenics under drought as previously reported. The model is further supported by down-regulation of several chloroplastidic *PPOs*, another major Cu protein, in the GS poplar during drought conditions. Our results suggest that alterations in N metabolism in GS transgenics cause differential regulation of genes involved in ROS protection under drought conditions leading to drought tolerance observed in the transgenics. Cu homeostasis and antioxidant regulation in response to altered N metabolism in the GS poplars need to be further investigated.

## Supporting Information

Figure S1 **Expression of poplar reference genes selected for RT-qPCR analysis across all tissues and conditions in the present study.** The three reference genes were selected according to Vandesompele et al. [40] and validated as reference genes: elongation factor 1β (EF1β), actin (ACT), and ubiquitin (UBQ). Samples for sets 1 and 2 are ordered as follows: sink leaf, source leaf, stem, main root and fine roots in well-watered, drought and recovery. Values for pairwise variation for the three reference genes (V_3_) considering their expression in all samples, were calculated using geNorm. V_3_ values obtained (0.098 and 0.13 for the first and second replicates, respectively) were lower than the cut-off (0.15) proposed by Vandesompele et al. [40]. Values are presented as quantitative cycles (C_q_) for each of the three reference genes.
(TIF)

Figure S2 **Similarity matrix for deduced** ***Arabidopsis*** **and poplar SOD amino acid sequences.** Similarities between protein sequences were calculated based on pairwise alignments using the EMBOSS Pairwise Alignment Algorithms (http://www.ebi.ac.uk/Tools/emboss/align/).
(TIF)

Figure S3 **Proposed exon sequences for** ***PtFSD2.2*** **after manual curation using the** ***PtFSD2.1*** **gene model found in Phytozome.** Nucleotide insertions are shown in shade. The premature stop codon is underlined in exon six.
(TIFF)

Figure S4 **Whole-genome microarray analysis (Agilent** ***Populus*** **whole genome array; 4x44K platform) of genes differentially expressed between wild type and GS transgenics.** Differential expression was determined by *p*-values adjusted with the SLIM method [74], with a fold-change cut-off of two. Relative expression (log ratio of GS/wild type) in four tissues [sink leaves (**SiL**), source leaves (**SoL**), stems (**Stm**) and main roots (**RA**)] during drought was shown, with red indicating up-regulation and blue, down-regulation in the GS transgenics. Two biological replicates were included. Genes annotated as superoxide dismutase are listed in bold.
(TIF)

Table S1 Proposed poplar SOD gene nomenclature based on SODs described for *Arabidopsis thallaina* [15] and forward and reverse primers used for RT-qPCR analysis.
(DOCX)

Table S2 Two-way ANOVA of observed transcript levels of SOD genes (all tissues vs. water availability) in wild type plants. Genes are sorted by P-values. Genes with P-values ≤0.05 appear in bold.
(DOCX)

Table S3 Two-way ANOVA of observed transcript levels of SOD genes between the two genotypes across all tissues in each growth condition. Genes are sorted by P-values. Genes with P-values ≤0.05 appear in bold.
(DOCX)
