# Supplementary figures and images for: The Populus Superoxide Dismutase Gene Family and Its Responses to Drought Stress in Transgenic Poplar Overexpressing a Pine Cytosolic Glutamine Synthetase (GS1a)
Source: PLoS One. 2013 Feb 22;8(2):e56421. doi: 10.1371/journal.pone.0056421 (PMC3579828; doi:10.1371/journal.pone.0056421)

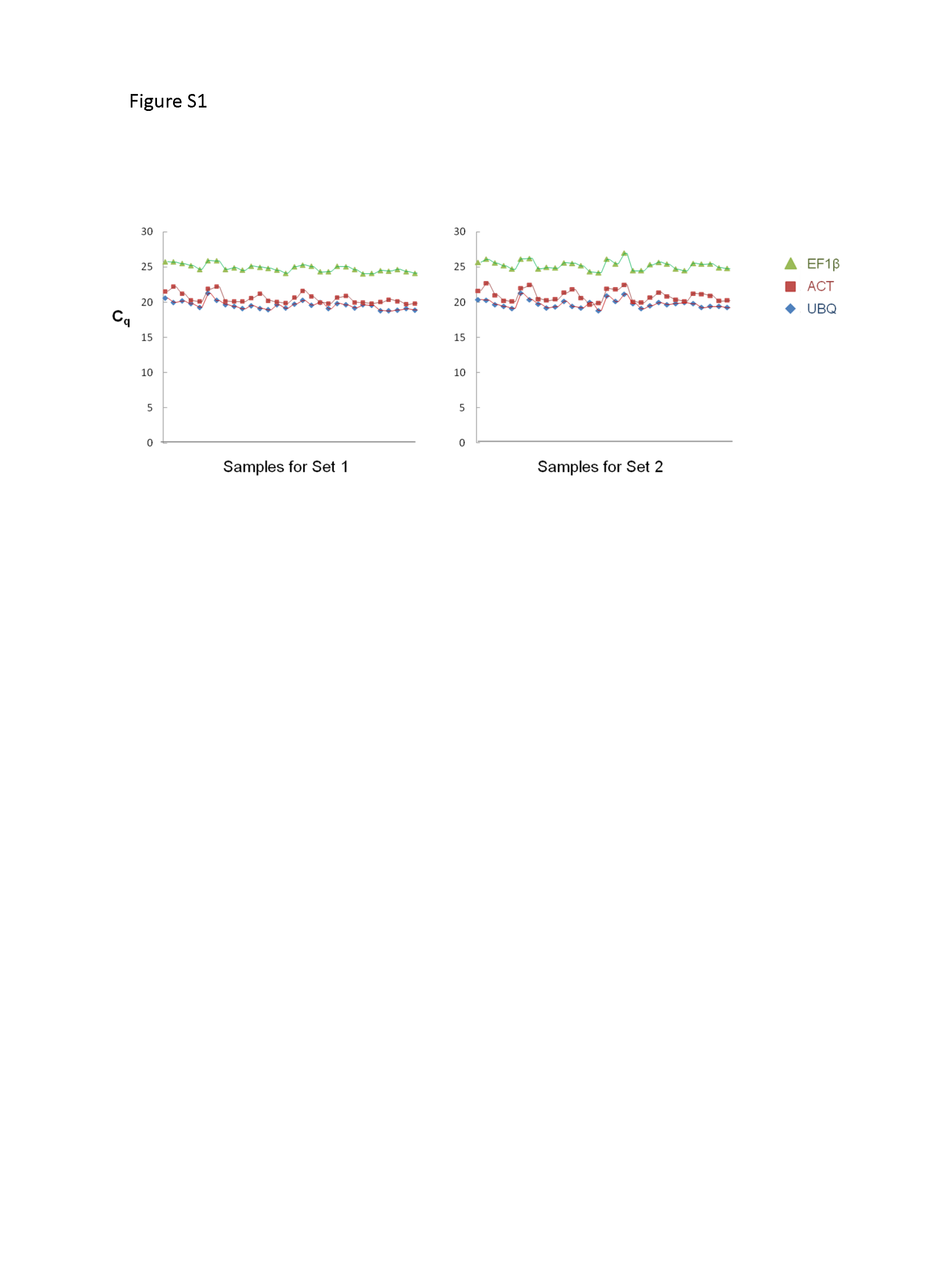

Supplement: Figure S1 — Expression of poplar reference genes selected for RT-qPCR analysis across all tissues and conditions in the present study. The three reference genes were selected according to Vandesompele et al. [40] and validated as reference genes: elongation factor 1β (EF1β), actin (ACT), and ubiquitin (UBQ). Samples for sets 1 and 2 are ordered as follows: sink leaf, source leaf, stem, main root and fine roots in well-watered, drought and recovery. Values for pairwise variation for the three reference genes (V3) considering their expression in all samples, were calculated using geNorm. V3 values obtained (0.098 and 0.13 for the first and second replicates, respectively) were lower than the cut-off (0.15) proposed by Vandesompele et al. [40]. Values are presented as quantitative cycles (Cq) for each of the three reference genes. (TIF) [file pone.0056421.s001.tif]

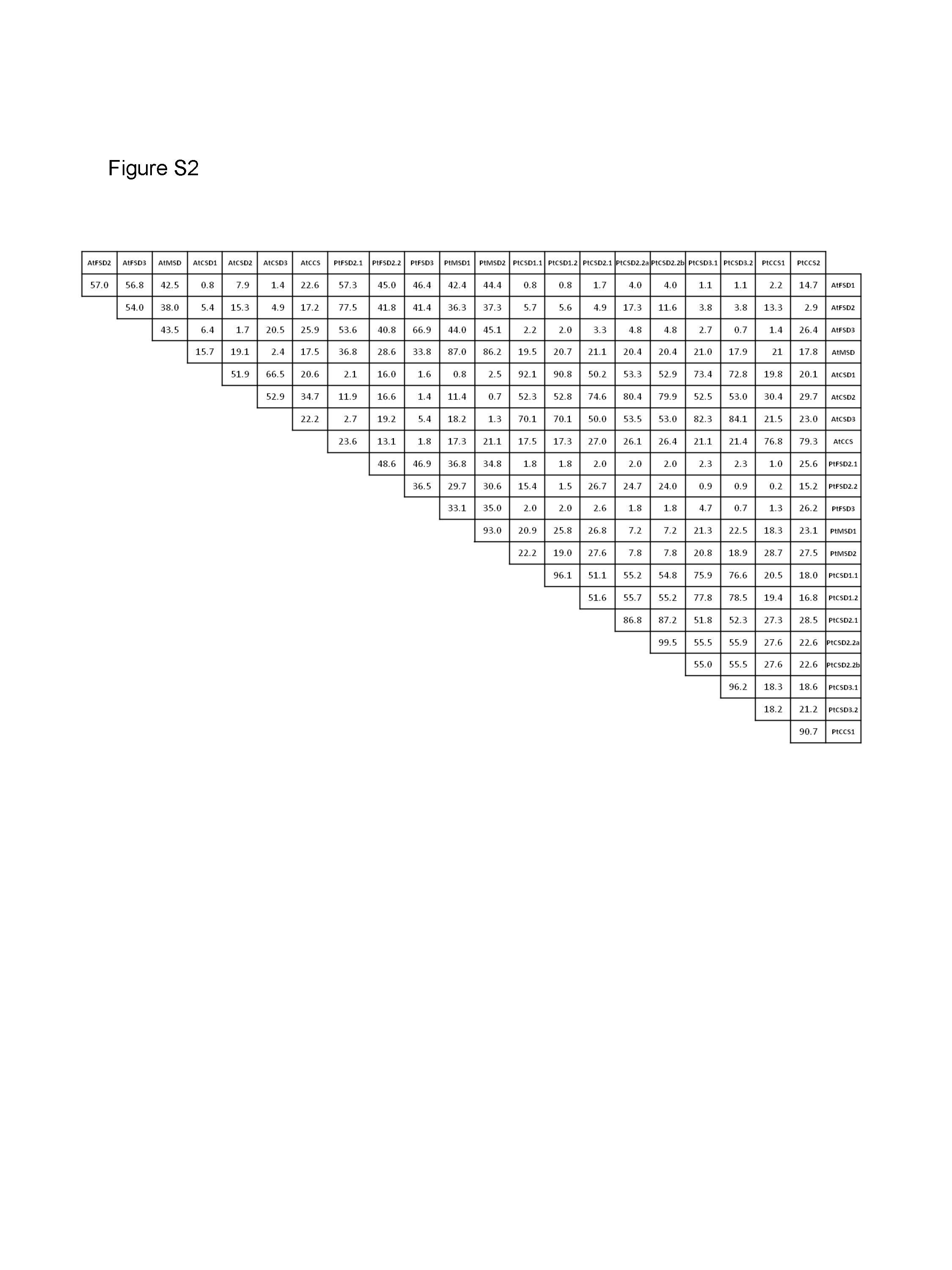

Supplement: Figure S2 — Similarity matrix for deduced Arabidopsis and poplar SOD amino acid sequences. Similarities between protein sequences were calculated based on pairwise alignments using the EMBOSS Pairwise Alignment Algorithms (http://www.ebi.ac.uk/Tools/emboss/align/). (TIF) [file pone.0056421.s002.tif]

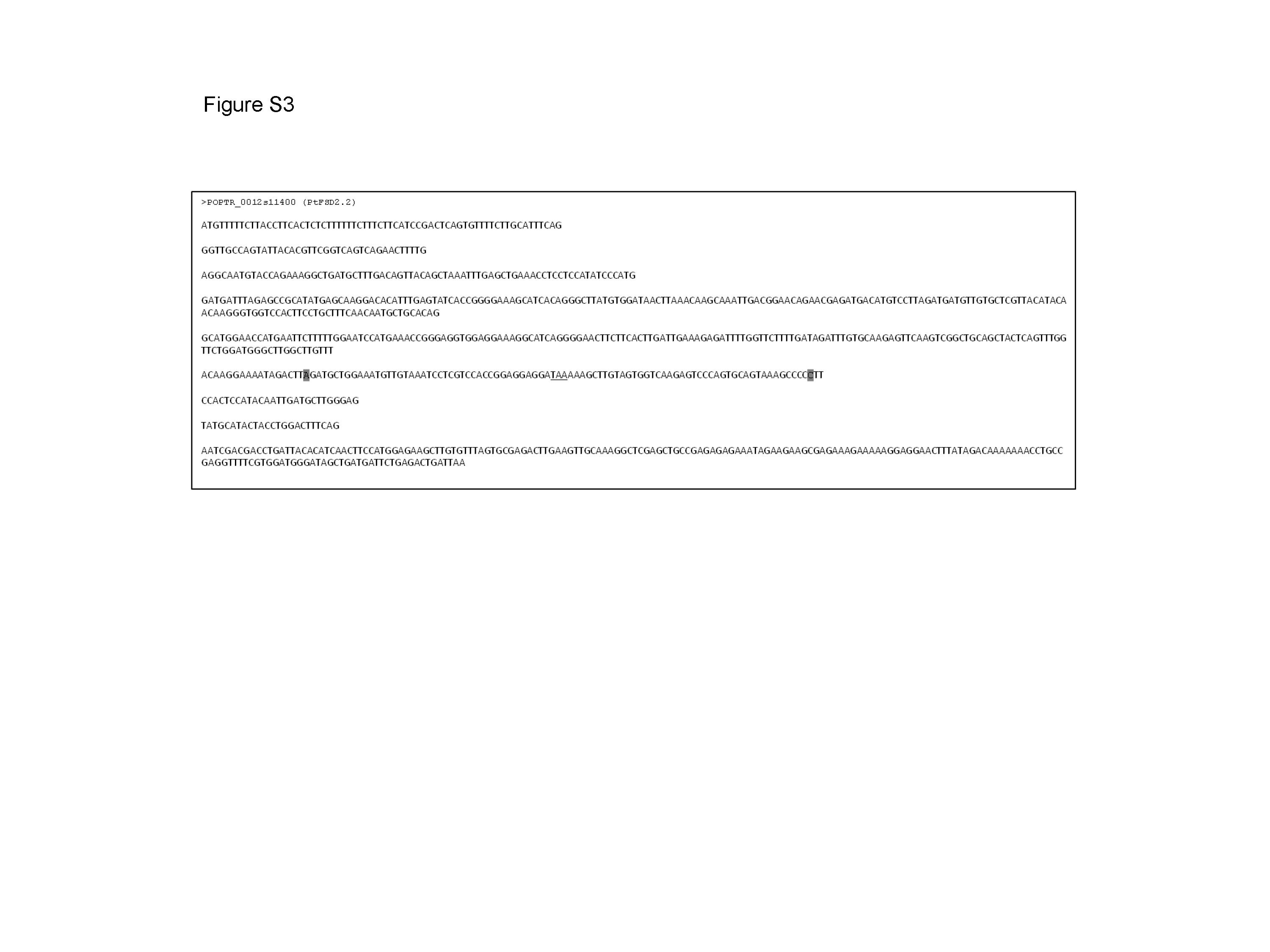

Supplement: Figure S3 — Proposed exon sequences for PtFSD2.2 after manual curation using the PtFSD2.1 gene model found in Phytozome. Nucleotide insertions are shown in shade. The premature stop codon is underlined in exon six. (TIFF) [file pone.0056421.s003.tif]

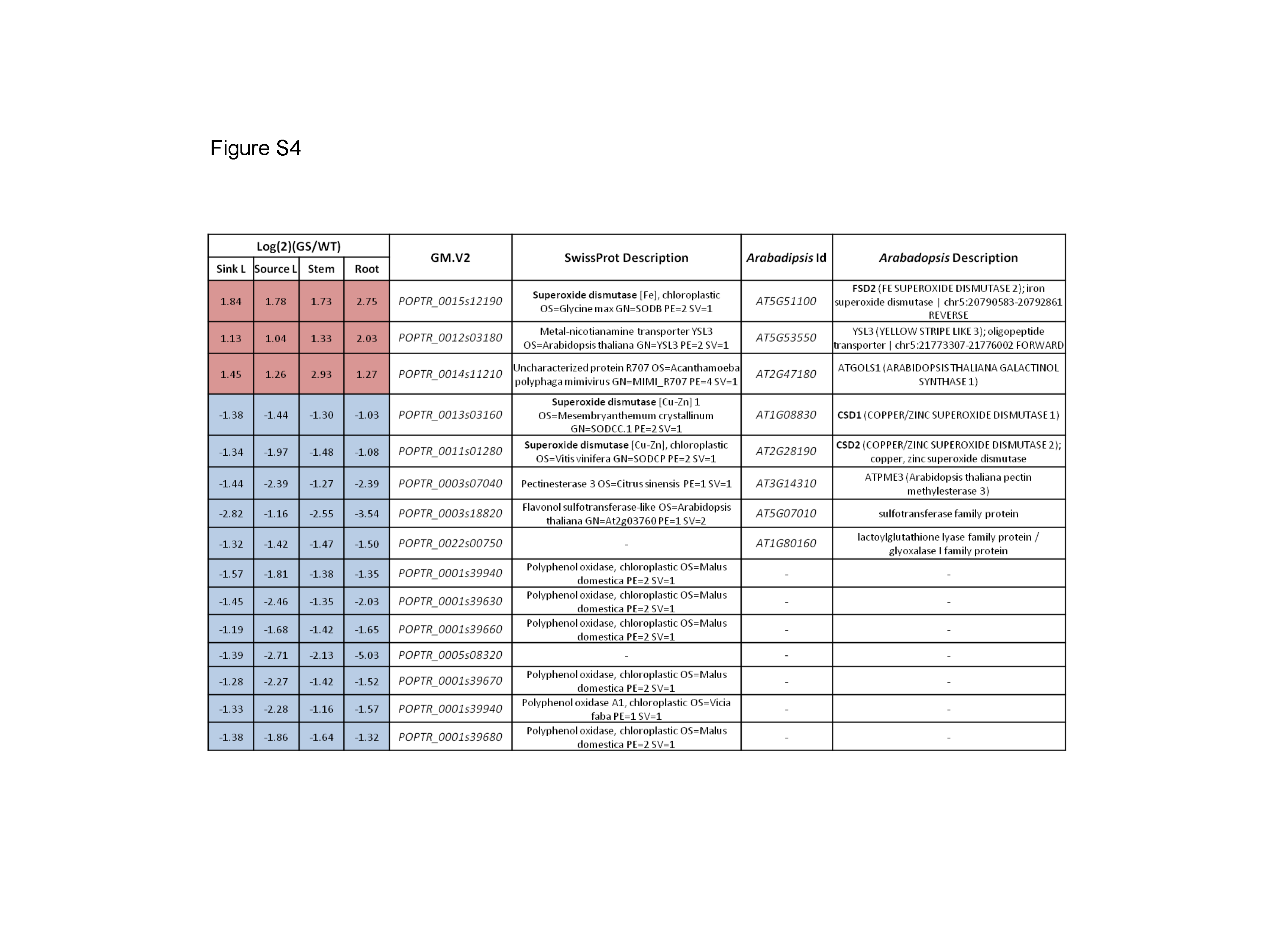

Supplement: Figure S4 — Whole-genome microarray analysis (Agilent Populus whole genome array; 4x44K platform) of genes differentially expressed between wild type and GS transgenics. Differential expression was determined by p-values adjusted with the SLIM method [74], with a fold-change cut-off of two. Relative expression (log ratio of GS/wild type) in four tissues [sink leaves (SiL), source leaves (SoL), stems (Stm) and main roots (RA)] during drought was shown, with red indicating up-regulation and blue, down-regulation in the GS transgenics. Two biological replicates were included. Genes annotated as superoxide dismutase are listed in bold. (TIF) [file pone.0056421.s004.tif]
