# Supplementary material for: The Populus Superoxide Dismutase Gene Family and Its Responses to Drought Stress in Transgenic Poplar Overexpressing a Pine Cytosolic Glutamine Synthetase (GS1a)
Source: PLoS One. 2013 Feb 22;8(2):e56421. doi: 10.1371/journal.pone.0056421 (PMC3579828; doi:10.1371/journal.pone.0056421)
Supplement: Table S1 — Proposed poplar SOD gene nomenclature based on SODs described for Arabidopsis thallaina [15] and forward and reverse primers used for RT-qPCR analysis. (DOCX) [file pone.0056421.s005.docx]

**Table S1.**

| ***Populus trichocarpa* SOD gene nomenclature** | **Locus** | **Forward (5’→3’)** | **Reverse(5’→3’)** |
| --- | --- | --- | --- |
| *PtCCS1* | *POPTR_0001s08330* | GCCAGAAGTGCAGGAGTTGG | CAGTGAACAGAGTAAAACAAACACAGAG |
| *PtCCS2* | *POPTR_0003s11830* | GCCAGAAGTGCAGGAGTTGG | CAATGGCTGAACATGGTGC |
| *PtCSD1.1* | *POPTR_0005s04590* | CTGTTGGTGATGATGGCACT | ACTATGGCGGTGCTGTGG |
| *PtCSD1.2* | *POPTR_0013s03160* | CTGTTGGTGATGATGGCACT | GGCTTTCATATTTTTATTCAGAATCTATC |
| *PtCSD2.1* | *POPTR_0009s01050* | ACTGGGAATGCAGGTGGA | CCTGATAGTATTACTTTACACACTGAGAA |
| *PtCSD2.2a* | *POPTR_0011s01280* | CACTGAGTGGTCCAAATGCA | CACATGGGAAAATTAACATTAACATTA |
| *PtCSD2.2b* | *POPTR_0004s22620* | as for *PtCSD2.2a* | as for *PtCSD2.2a* |
| *PtCSD3.1* | *POPTR_0019s05140* | AGACAACTGGGAATGCAGGT | CAGTTTCGAACAAGTATATTGGATC |
| *PtCSD3.2* | *POPTR_0013s05350* | AGACAACTGGGAATGCAGGT | GCACAAGTGTGTTGGACGAG |
| *PtMSD1* | *POPTR_0019s08540* | CAAGCACACCTGCTCTGCA | TTTCCATAGTTTCGATACACCAGTAA |
| *PtMSD2* | *POPTR_0013s09270* | GGTGAAGTTTATGACAAAGAAAGC | TAACATCCAACGAACCACGG |
| *PtFSD2.1* | *POPTR_0015s12190* | TGGTGTCATGGGATGCAG | AAGACAACGAAGGACGTGACA |
| *PtFSD2.2* | *POPTR_0012s11400* | CACATCAACTTCCATGGAGAA | CCGATGCCTGGATATTCATG |
| *PtFSD3* | *POPTR_0005s09190* | GCAGAGGCATTCGTGAATCT | CCAACATGACTGCATTTCTACC |
|  |  |  |  |
| ***Arabidopsis thaliana* SOD gene nomenclature** | **Locus** |  |  |
| *AtCCS* | *AT1G12520* |  |  |
| *AtCDS1* | *AT1G08830* |  |  |
| *AtCSD2* | *AT2G28190* |  |  |
| *AtCSD3* | *AT5G18100* |  |  |
| *AtMSD* | *AT3G10920* |  |  |
| *AtFSD1* | *AT4G25100* |  |  |
| *AtFSD2* | *AT5G51100* |  |  |
| *AtFSD3* | *AT5G23310* |  |  |
