# Supplementary material for: The Populus Superoxide Dismutase Gene Family and Its Responses to Drought Stress in Transgenic Poplar Overexpressing a Pine Cytosolic Glutamine Synthetase (GS1a)
Source: PLoS One. 2013 Feb 22;8(2):e56421. doi: 10.1371/journal.pone.0056421 (PMC3579828; doi:10.1371/journal.pone.0056421)
Supplement: Table S2 — Two-way ANOVA of observed transcript levels of SOD genes (all tissues vs. water availability) in wild type plants. Genes are sorted by P-values. Genes with P-values ≤0.05 appear in bold. (DOCX) [file pone.0056421.s006.docx]

| **Well-watered *vs*. Drought** | |  | **Well-watered *vs*. Recovery** | |
| --- | --- | --- | --- | --- |
| *gene* | P-value |  | *gene* | P-value |
| ***PtFSD2.1*** | **3.65091E-06** |  | ***PtCCS1*** | **1.20013E-05** |
| ***PtCSD1.1*** | **0.001912709** |  | ***PtCSD3.2*** | **0.000202235** |
| ***PtCSD3.2*** | **0.006035689** |  | ***PtMSD1.1*** | **0.001477703** |
| ***PtCSD2.2*** | **0.006964789** |  | ***PtFSD2.2*** | **0.013692673** |
| ***PtCCS1*** | **0.007220917** |  | ***PtCSD2.2*** | **0.025642525** |
| ***PtMSD1.1*** | **0.013302107** |  | ***PtMSD1.2*** | **0.042785742** |
| ***PtMSD1.2*** | **0.013794331** |  | *PtCSD3.1* | 0.053872381 |
| ***PtCSD1.2*** | **0.028987756** |  | *PtCSD2.1* | 0.056012715 |
| ***PtCCS2*** | **0.029695719** |  | *PtFSD3* | 0.071263661 |
| ***PtCSD3.1*** | **0.042395926** |  | *PtFSD2.1* | 0.324155133 |
| *PtFSD3* | 0.069425621 |  | *PtCCS2* | 0.419061163 |
| *PtCSD2.1* | 0.704250451 |  | *PtCSD1.2* | 0.644898431 |
| *PtFSD2.2* | 0.742713244 |  | *PtCSD1.1* | 0.751614377 |

**Table S2.**
