# Supplementary material for: The Populus Superoxide Dismutase Gene Family and Its Responses to Drought Stress in Transgenic Poplar Overexpressing a Pine Cytosolic Glutamine Synthetase (GS1a)
Source: PLoS One. 2013 Feb 22;8(2):e56421. doi: 10.1371/journal.pone.0056421 (PMC3579828; doi:10.1371/journal.pone.0056421)
Supplement: Table S3 — Two-way ANOVA of observed transcript levels of SOD genes between the two genotypes across all tissues in each growth condition. Genes are sorted by P-values. Genes with P-values ≤0.05 appear in bold. (DOCX) [file pone.0056421.s007.docx]

**Table S3.**

|  | Wild Type versus GS transgenic | | | | | | |
| --- | --- | --- | --- | --- | --- | --- | --- |
|  | Well-watered |  |  | Drought |  |  | Recovery |
| *gene* | P-value |  | *gene* | P-value |  | *gene* | P-value |
| ***PtFSD2.1*** | **0.000104018** |  | ***PtFSD2.1*** | **6.42683E-08** |  | ***PtFSD2.1*** | **0.00246786** |
| ***PtCSD1.1*** | **0.007607798** |  | ***PtCSD2.2*** | **4.52387E-05** |  | ***PtCSD2.1*** | **0.012786695** |
| *PtCSD2.1* | 0.129594864 |  | ***PtCCS1*** | **0.00097276** |  | ***PtCSD2.2*** | **0.015633634** |
| *PtFSD3* | 0.269570314 |  | ***PtCSD1.1*** | **0.001123206** |  | *PtMSD1.2* | 0.079931607 |
| *PtMSD1.1* | 0.37637595 |  | ***PtCCS2*** | **0.001362754** |  | *PtCSD3.1* | 0.115612771 |
| *PtCSD3.2* | 0.420009982 |  | ***PtCSD2.1*** | **0.004586284** |  | *PtCSD1.1* | 0.179683425 |
| *PtCSD2.2* | 0.499305768 |  | ***PtMSD1.2*** | **0.011162315** |  | *PtCCS2* | 0.22688548 |
| *PtMSD1.2* | 0.574089753 |  | ***PtCSD3.2*** | **0.021650143** |  | *PtCSD3.2* | 0.322669605 |
| *PtCCS1* | 0.67187502 |  | *PtCSD3.1* | 0.057296602 |  | *PtMSD1.1* | 0.458246581 |
| *PtCSD3.1* | 0.896031442 |  | *PtMSD1.1* | 0.111755921 |  | *PtCCS1* | 0.642570843 |
| *PtCCS2* | 4.964602744 |  | *PtFSD3* | 0.144947983 |  | *PtFSD3* | 0.822094614 |
